# A Case of Persistent Hiccup after Laparoscopic Cholecystectomy

**DOI:** 10.1155/2013/206768

**Published:** 2013-04-04

**Authors:** Elisa Grifoni, Costanza Marchiani, Alessia Fabbri, Gabriele Ciuti, Andrea Pavellini, Francesco Mancuso, Riccardo Viligiardi, Alberto Moggi Pignone

**Affiliations:** ^1^SOD Medicina Interna ad Orientamento all'Alta Complessità Assistenziale 3, AOU Careggi, Largo Brambilla 3, 50134 Firenze, Italy; ^2^SOD Chirurgia Generale e di Urgenza 1, AOU Careggi, Largo Brambilla 3, 50134 Firenze, Italy

## Abstract

A 79-year-old man, with history of recent laparoscopic cholecystectomy, came to our attention for persistent hiccup, dysphonia, and dysphagia. Noninvasive imaging studies showed a nodular lesion in the right hepatic lobe with transdiaphragmatic infiltration and increased tracer uptake on positron emission tomography. Suspecting a malignant lesion and given the difficulty of performing a percutaneous transthoracic biopsy, the patient underwent surgery. Histological analysis of surgical specimen showed biliary gallstones surrounded by exudative inflammation, resulting from gallbladder rupture and gallstones spillage as a complication of the previous surgical intervention. This case highlights the importance of considering such rare complication after laparoscopic cholecystectomy.

## 1. Introduction

Since its introduction, laparoscopic cholecystectomy has become the gold standard of treatment for symptomatic gallstone disease. Gallbladder perforation by trocars with gallstone spillage is an extremely rare event, which can lead to several complications, such as abdominal wall and intraabdominal abscesses formation [1]. 

## 2. Case Presentation

**Figure 1:**
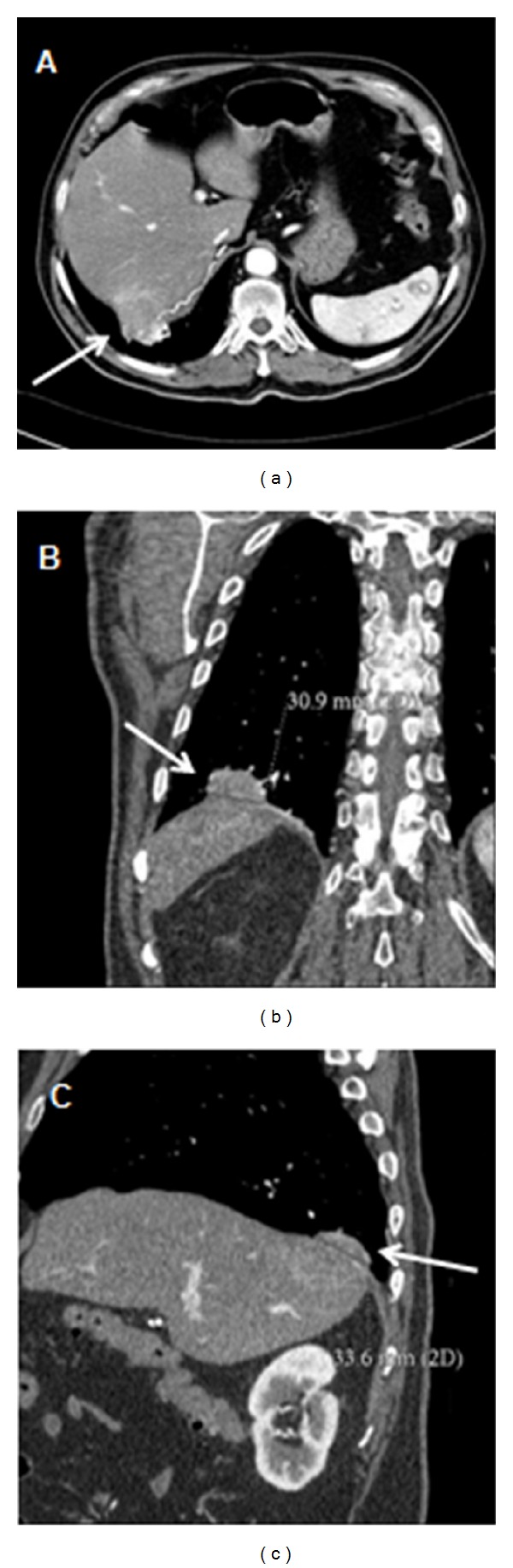
Axial (a), coronal (b), and sagittal (c) images from a computed tomography of neck, thorax, and abdomen with intravenous contrast demonstrating a nodular image close to the diaphragm, in the right side, with transdiaphragmatic infiltration, hepatic invasion, and arterial vascularization (*arrows*).

**Figure 2:**
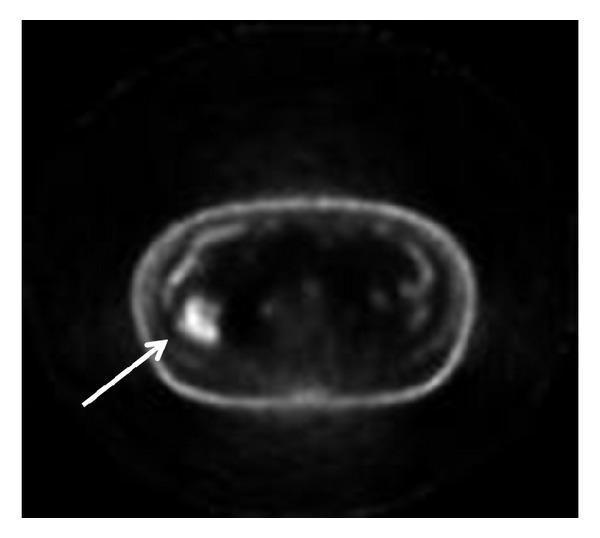
Positron emission tomography scan showing an area of increased tracer uptake in the right costal-phrenic angle (*arrow*).

**Figure 3:**
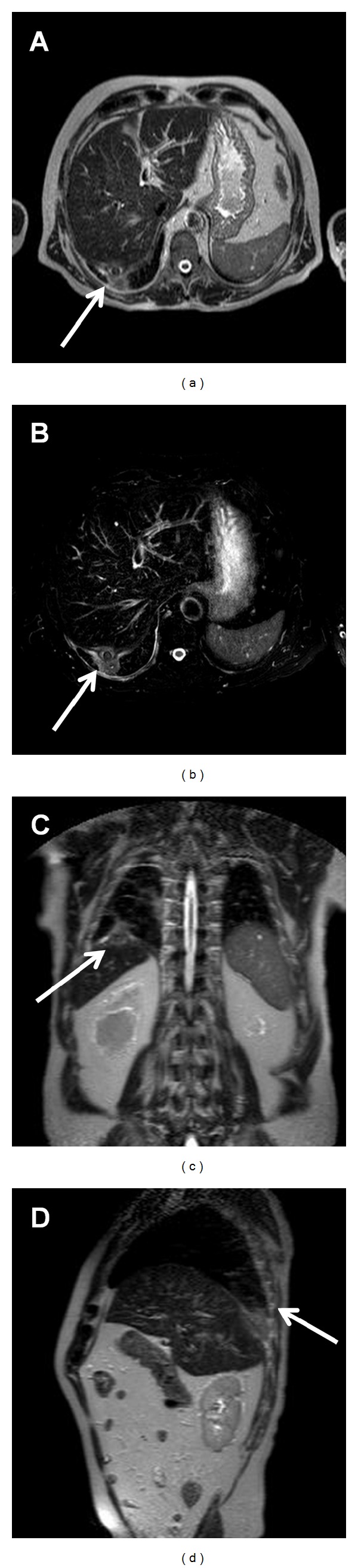
Axial (a and b), coronal (c) and sagittal (d) images from an abdomen magnetic resonance showing a lesion with solid component surrounded by a blood-like fluid labrum, with compression of the underlying liver parenchyma, pulmonary consolidation and minimal pleural effusion (*arrows*).

A 79-year-old man, former smoker, with history of arterial hypertension, atrial fibrillation, and laparoscopic cholecystectomy for gallstone disease 4 months before, came to our attention for the onset of hiccup, dysphonia, and dysphagia for one month. Suspecting a gastroesophageal reflux, the patient had been initially treated with proton pump inhibitors for 2 weeks but without symptoms relief. Physical examination was normal; in particular, no abnormal masses, lymphadenopathies, or signs of mediastinal involvement were present. Blood tests revealed normocytic hypochromic hyposideremic anemia with positivity of faecal occult blood test, increase of neuron specific enolase (NSE), cromogranin A, and beta2-microglobulin. Chest X-ray did not show abnormal findings. Abdominal ultrasound revealed a subcapsular hypoechoic mass of about 42 × 31 mm in the right hepatic lobe. Computed tomography of neck, thorax, and abdomen with intravenous contrast demonstrated a nodular image of about 25 × 37 mm close to the diaphragm, in the right side, with transdiaphragmatic infiltration and invasion of the 7th hepatic segment, contrastographic enhancement of the diaphragm in the same location, and arterial vascularization (Figure 1). Small subcarinal lymph nodes were present. Positron emission tomography showed areas of increased tracer uptake in the right costal-phrenic angle and mediastinal subcarinal region (Figure 2). An abdomen magnetic resonance further characterized the lesion, showing a solid component surrounded by a blood-like fluid labrum, with compression of the underlying liver parenchyma, pulmonary consolidation, and minimal pleural effusion close to the lesion (Figure 3). For positivity of faecal occult blood test esophago-gastro-duodenal and colon endoscopy were also performed; they showed only telangiectasia of the inferior part of esophagus and sigma diverticulosis, respectively. No biopsies were performed due to the difficulty of a percutaneous transthoracic approach. Therefore, suspecting a neoplastic lesion, the patient underwent surgery via a left thoracoabdominal approach. An oval lesion of about 30 mm of diameter infiltrating the diaphragm and closely adherent to the lung was enucleated. The histological analysis of surgical specimen showed biliary gallstones surrounded by exudative inflammation with foreign body giant cells. The patient quickly recovered from surgical intervention with complete symptom relief.

## 3. Discussion

Since its introduction, laparoscopic cholecystectomy has become the gold standard of treatment for symptomatic gallstone disease with morbidity rates ranging from 2% to 11% compared to 4%–6% for elective open cholecystectomy. The benefits of laparoscopic cholecystectomy for gallbladder surgery are significant, minimizing mortality rates in the perioperative period, reducing the length of stay in the hospital, and allowing patients to return to their normal activities sooner when compared to open cholecystectomy. However, it carries some major complications, such as damage to the biliary system, blood vessels, and gastrointestinal tract. Additionally, gallbladder perforation by trocars in laparoscopic cholecystectomy leads to bile and gallstone spillage with a reported incidence of 5.4%–19% [1]. Theoretically, spilled gallstones can be displaced to any part of the abdominal cavity leading to several complications, such as abdominal wall and intraabdominal abscesses. The intraabdominal abscesses are commonly located in the subhepatic space or in its retroperitoneal region. Fistula formations, hernia sacs, and ovary and fallopian tubes containing lost gallstones are among some of the rare complications noted for gallstone abscess. Migration of stones through the diaphragm has been also described in case reports, mainly secondary to subphrenic abscesses. The underlying pathophysiologic process involves inflammatory reaction secondary to the presence of retained stones. They may then erode through the diaphragm and cause a bronchopleural fistula with cholelithopthysis, thoracic empyema, or pulmonary abscess [2–4]. Abscess formation from dropped stones after laparoscopic cholecystectomy has been reported to have an average duration of 4 months, like in our case, to 10 years [5]. In our case, noninvasive imaging studies initially supported the hypothesis of a neoplastic lesion. Secondary involvement of the diaphragm from other intraabdominal or intrathoracic tumors can occur, commonly by direct extension, from mesothelioma, lung cancer, and hepatic carcinoma. Instead, primary tumors of the diaphragm are rare and more than half are benign. The most common benign lesions are bronchogenic or mesothelial cysts and lipoma, while the most common primary malignant lesion is rhabdomyosarcoma [6]. The inflammatory reaction secondary to the presence of retained gallstones can simulate proliferative lesions, giving false positive results on imaging studies such as positron emission tomography. This case highlights the importance of considering such rare but possible complication accompanying gallbladder surgery in patients with history of laparoscopic cholecystectomy.
